# Housing of Cull Sows in the Hours before Transport to the Abattoir—An Initial Description of Sow Behaviour While Waiting in a Transfer Vehicle

**DOI:** 10.3390/ani7010001

**Published:** 2016-12-22

**Authors:** Mette S. Herskin, Katrine K. Fogsgaard, Ditte Erichsen, Mia Bonnichsen, Charlotte Gaillard, Karen Thodberg

**Affiliations:** Department of Animal Science, AU-Foulum, Aarhus University, DK-8830 Tjele, Denmark; Katrine.kopfogsgaard@anis.au.dk (K.K.F.); Charlotte.gaillard@anis.au.dk (C.G.); Karen.thodberg@anis.au.dk (K.T.)

**Keywords:** behaviour, welfare, cull sows, animal transport, pre-slaughter logistic chain

## Abstract

**Simple Summary:**

In modern pig production, sows are transported by road to abattoirs. However, for reasons of biosecurity, commercial trucks may have limited access to farms. This study described behaviour of sows while waiting for a commercial truck in transfer vehicles near a public road, as is common practice in Denmark. The study involved 106 sows from 11 loads. The sows stayed in the transfer vehicles for 6–59 min. In this period, the behaviour of the sows was characterised by aggression and only very limited resting. These preliminary results suggest that a pre-transport stay in a transfer vehicle can be challenging for sow welfare, especially for longer stays and during hot days.

**Abstract:**

In modern pig production, sows are transported by road to abattoirs. For reasons of biosecurity, commercial trucks may have limited access to farms. According to Danish regulations, sows can be kept in stationary transfer vehicles away from the farm for up to two hours before being loaded onto the commercial truck. We aimed to describe the behaviour of sows in transfer vehicles. This preliminary, exploratory study included data from 11 loads from a total of six Danish sow herds. Selection of animals to be slaughtered was done by the farmers. Clinical registrations were made before collection of the sows, after which they (in groups of 7–13) were mixed and moved to the transfer vehicle (median stocking density: 1.2 sow/m^2^), and driven a short distance to a public road. The duration of the stays in the transfer vehicles before being loaded onto the commercial trucks ranged from 6–59 min. During this period, the median frequency of aggressive interactions per load was 18 (range: 4–65), whereas the median frequency of lying per load was 1 (range: 0–23). The duration of the stay correlated positively with the frequency of aggressive interactions (r_s_ = 0.89; *n* = 11; *p* < 0.001) and with the frequency of lying (r_s_ = 0.62; *n* = 11; *p* < 0.05). Frequency of aggressive interactions correlated positively with the temperature inside the transfer vehicle (r_s_ = 0.89; *n* = 7; *p* < 0.001). These preliminary results are the first to describe the behaviour of cull sows during waiting in transfer vehicles, and may suggest that this period can be challenging for sow welfare, especially for longer stays and during hot days.

## 1. Introduction

In modern pig production, sows are transported by road to abattoirs after their productive lives. However, for reasons of biosecurity (in order to limit the risk of spreading diseases), commercial pig trucks may have limited access to farms. Hence, one extra link in the pre-slaughter logistic chain [1]—the transfer vehicle—has been added. According to Danish regulations [2,3], which are aggravating compared to European regulations [4], cull sows cannot be transported by commercial trucks for more than eight hours, but may be kept by the farmer in stationary transfer vehicles away from the farm for up to two hours before being loaded onto the commercial transport truck. Only limited research has focused on cull sows and their welfare. As discussed by de Jong et al. [5], reasons for culling are influenced by factors such as sow genotype, housing conditions and management policies. However, recent studies focusing on reports from farmers, such as Zhao et al. [6], found that almost 80% of the sows were not culled as part of a strategy, and that the vast majority of them were culled due to reproductive problems or reduced health. Similar conclusions have been drawn from studies involving clinical examination of sows upon arrival at commercial slaughterhouses in Europe [5] and the U.S. [7]. By studying the interval from insemination to culling, de Hollander et al. [8] showed that a large proportion of sows were culled after weaning, and similar findings were reported by Engblom et al. [9]. Recently, McGee et al. [10] showed that after unloading at U.S. buying stations, cull animals made up the majority of fatigued and lame pigs, and consequently suggested that these animals may be less fit for transport than slaughter pigs. Similar suggestions have been put forward by Nielsen et al. [11]. 

Such suggestions raise questions regarding the vulnerability of this particular group of animals when exposed to challenges such as mixing, crowding or lack of possibility to thermoregulate—which they most likely will face, when transfer vehicles are being used. Recent recommendations from the World Organisation for Animal Health (OIE) [12] underline the possible vulnerability of cull sows and state that, for example, old, lactating and aggressive animals require special conditions on the day of slaughter in order to ensure their welfare. Despite these potential negative consequences of a stay in a transfer vehicle, to date, no studies have focused on this management procedure—a procedure which may be advantageous from a biosecurity point-of-view, but potentially affect the welfare of the sows, as well as their fitness for the planned transport to slaughter in a negative way. 

In this preliminary, explorative study, we aimed to describe the behaviour of sows in transfer vehicles. We included data from 11 loads of sows from a total of six Danish sow herds, collected as part of a larger project on fitness for transport in cull sows [13,14]. We hypothesized that the time spent in a transfer vehicle would be characterised by aggression and lack of resting, and that increasing temperature inside the vehicles would be associated with increased aggression due to the limited possibility to thermoregulate. 

## 2. Materials and Methods

### 2.1. Experimental Design

This observational study involved 106 sows from six commercial Danish sow herds. Sow herds were included based on the distance to one of the larger Danish slaughterhouses [13,14]. Within four distance categories, a random postal code was selected, and randomly chosen farmers within the postal code, sending more than an average of 8 sows per load, were contacted by phone. Herds could be included if they were willing to participate, and if they sent sows to the specific slaughterhouse. For the present dataset, inclusion further required the use of a transfer vehicle. Data was collected during 2015 and included data from 11 loads visiting a total of six Danish sow herds. 

Selection of animals to be slaughtered and thus included in the dataset was done by the farmers. For ethical and legal reasons, unfit sows (as described in Chapter 1 of Annex 1 of [4]) could not be included in the dataset. In the hours before loading onto the transfer vehicle, all sows chosen by the farmer were examined clinically. All experimental procedures were approved by the Danish Animal Experiments Inspectorate (permit no. 2015-15-0201-00715). The ethical permit allowed the disregarding of special Danish regulations for fitness for transport.

### 2.2. Procedures

Before loading onto the transfer vehicles, the sows were kept, fed and managed according to Danish commercial practice. After the clinical registrations, the sows to be slaughtered on a specific day and farm were taken from their home pen or stall and loaded onto the transfer vehicle by farm staff, where they were mixed (in groups of 7–13 animals; median group size 9.0), driven a short distance to a public road and left there waiting for the commercial truck. The involved transfer vehicles, their design and the availability of resources were chosen by the farmers, and used repeatedly when herds participated more than once in the study. The available area in the transfer vehicles ranged from 7.2 to 20 m^2^, resulting in a stocking density of 0.4–1.8 sows/m^2^ (median stocking density: 1.2 sows/m^2^). The sows were marked individually with colour spray. The duration of the stays in the transfer vehicles before being loaded unto the commercial trucks ranged from 6–59 min (median duration: 21 min), and was terminated when the commercial truck arrived to bring the sows to slaughter.

### 2.3. Data Collection

The clinical examination of the sows before loading them onto the transfer vehicles consisted of several parameters. For the present study, recordings of lameness score (on a scale from 0–3, where 0: normal gait; 1: abnormal gait, all legs are weight bearing; 2: lame to a degree where the affected limb can be recognised, the use of the limb is limited; and 3: seriously lame, does not bear weight on the affected limb or avoids walking), body condition score (using a ½-point scale from 1–4 focusing on the visibility of ribs, spine and hips and 1: skinny (can be recognised visually); 2: slim (requires light pressure to recognise); 3: intermediate (requires hard pressure to recognise) and 4: fat (cannot be seen or felt)) and presence of milk in the udder (assessed as 0/1 by udder palpation) were made. 

Before the sows were loaded onto a transfer vehicle, one temperature logger (iButton DS1923, Maxim Integrated, San Jose, CA, USA; resolution ≤ 0.5 °C) was placed inside each vehicle at a height of approximately 1.1 m. The temperature inside the vehicle was logged every minute. After arrival of the commercial pig truck, the logger was taken down and data collected. 

During the stay in the transfer vehicle, behaviour of the sows was recorded by a hand-held video camera (Canon Legria HF R56, Canon, Diegem, Belgium), from outside the vehicle and at a distance of at least 1.2 m from the animals. The person filming took care not to disturb the animals. 

The behaviour of the sows was analysed by behaviour sampling and continuous recording [15]. One observer, blind to the experimental hypotheses, performed all behavioural analyses. The duration and frequency of two behavioural elements were recorded per load of sows: lying (a sow was scored as lying when her legs were not bearing weight) and aggressive interactions (including uni- as well as bi-directional interactions between at least two sows involving bites and/or head knocks). The following variables were calculated per load: (1) the number of observations of sows changing posture from active to lying; (2) the frequency of observations of posture change from active to lying per minute; (3) the number of aggressive interactions; and (4) the frequency of aggressive interactions per minute. 

### 2.4. Statistical Analyses

Descriptive statistics was used to describe the behaviour of the sows during the stays in the transfer vehicles. In addition, possible correlations between behavioural variables, the duration of the stay and the temperature in the vehicles were calculated using Spearman correlations. All statistical analyses were performed with SAS Enterprise Guide software (version 5.1, SAS Institute Inc., Cary, NC, USA). Results are presented per load and as medians across loads, except for temperature in the transfer vehicles, which is presented as mean ± STD per load. A probability level of *p* < 0.05 was considered statistically significant, whereas 0.05 < *p* < 0.10 was considered a tendency.

## 3. Results

### 3.1. Clinical Condition of the Cull Sows

The lameness score of the cull sows in this study ranged from 0–2 with a median of 0. The median body condition score was 3.5 (range 2–4). Among the 106 cull sows, 49% had milk in the udder.

### 3.2. Temperature in the Transfer Vehicles

Due to technical difficulties, temperature was only recorded for seven of the 11 involved loads. During the stays in the transfer vehicles, the temperature ranged from 4.6 to 28.1 °C. The mean temperature per load ranged from 5.3–26.3 °C (Table 1). 

### 3.3. Provision of Resources in the Transfer Vehicles

For each load of sows, Table 1 shows whether the floor of the transfer vehicle was provided with bedding (sawdust was provided for 2/9 loads), whether the transfer vehicle was provided with a roof (2/11 had a roof or were closed, the rest were open) and whether the sows had access to water during the stays in the transfer vehicles (none had access to water). 

### 3.4. Behaviour of the Sows during the Stay in the Transfer Vehicles

The behaviour of the cull sows from the 11 loads, as well as the medians across the loads, are shown in Table 1. 

### 3.5. Correlations between Behaviour, Duration of Stay and Temperature in the Transfer Vehicles

Significant positive correlations were found between the duration of the stay in the transfer vehicles and the occurrence of aggressive interactions (r_s_ = 0.89; *n* = 11; *p* < 0.001) and with the number of observations of sows changing posture from active to lying (r_s_ = 0.62; *n* = 11; *p* < 0.05). The occurrence of aggressive interactions correlated positively with the temperature inside the transfer vehicle (r_s_ = 0.89; *n* = 7; *p* < 0.001). 

## 4. Discussion

The present study is among the first to focus on cull sow behaviour and welfare, and aimed to make a preliminary description of the behaviour of cull sows from commercial Danish herds while they werewaiting in transfer vehicles before transport to an abattoir. The observational study included recordings of sow behaviour (lying and aggression) as well as the temperature in the transfer vehicles and the duration of the stays. Despite the variable conditions and durations of the studied stays in the transfer vehicles, in addition to the lack of control in the study, the results suggest that aggressive interactions are common and that resting is limited, which might render a stay in a transfer vehicle before transport to the abbatoir a challenge for cull sow welfare, especially for longer stays and during hot days. 

The behaviour of cull sows has received very limited scientific attention. Based on knowledge about the behaviour of sows in the minutes and hours after mixing [16,17], we hypothesized that a stay in a transfer vehicle would be characterised by a high level of aggressive interactions between the cull sows, which most likely were not familiar with each other, even though they came from the same herd. The results showed that aggressive interactions were observed for all 11 loads, even the ones lasting only a few minutes. Overall, the median occurrence of aggressive interactions per minute was 0.75 and the occurrence of aggressive interactions correlated positively with the duration of the stay. This last finding is not surprising considering the reported short latency to initiation of fights and the high frequency of aggressive interactions on the first day after mixing of sows [18], topped up by the rather high stocking density in the transfer vehicles compared to the normal on-farm conditions for group-housed sows. The consequences of mixing unfamiliar sows in terms of aggression have long been recognised as a welfare problem [19]. Especially for longer stays (up to the legal maximum of two hours) and on hot days, the high level of aggressive interactions, combined with the lack of supervision of the sows (supervision was recommended by [20]), which characterised all 11 loads, might have posed a welfare challenge, as well as a risk of lowered fitness for transport. 

In the present study, the stocking density ranged from 0.4–1.8 sows/m^2^ with a median of 1.2. As reviewed by Greenwood et al. [19], lack of space leads to increased incidence of aggressive interactions between sows. Several studies have provided recommendations for the stocking density of sows kept in groups (such as [21,22]), and they all exceeded the space provided to the sows in the present transfer vehicles. However, so far, no studies have dealt with the need for space in transfer vehicles or trucks for cull sows. Future studies should examine whether changes in the stocking density, provision of bedding or design of the available floor space as such, might be used to limit the occurrence of aggression among sows, thereby potentially limiting the impact of pre-slaughter stress on the animals. 

Despite a large variation among the present observations, the median number of occurrences of lying across the 11 loads was only one, and, for four out of 11 loads, no sows were observed to lie down. Taking the short duration of the stays on the transfer vehicles into account, this lack of resting behaviour was probably not a welfare problem for the sows. However, as compared to a proportion of 80% lying reported from on-farm sows [23], the stays in the transfer vehicles were characterised by markedly less resting. Even though the stays in the transfer vehicles were relatively short (less than one hour in the present data set), it can be discussed whether the conditions lived up to the recent recommendations from the OIE on pre-journey assembly areas allowing animals to rest [12]. 

As reported by recent studies [5,6,10], sows are often culled due to reproductive problems or reduced health. Recently, Ison et al. [24] have shown that primiparous sows show behavioural and physiological responses to mixing with older, unfamiliar sows. In general, primiparous sows will probably have a low representation of sows to be culled, but other vulnerable groups—such as weak or sick sows—may experience similar negative consequences when mixed with other sows before or during transport to abattoirs. In the present study, no knowledge was available regarding the reasons for culling, and it is therefore not possible to examine whether weaker animals responded differently to the stay in the transfer vehicles than the stronger ones. The available clinical data showed that approximately half of the cull sows were weaned recently, that they had only limited problems with lameness, and that their body condition score was high. Hence, the sows in the present study were probably in a relatively good condition. Future studies, focusing on fitness for transport in cull sows, should include effects of the clinical condition of the animals before loading onto transfer vehicles and the commercial trucks.

According to Danish regulations [2,3], cull sows may be kept in transfer vehicles for up to two hours, and this period is not counted in the duration of the later transport to the abattoir. Experiencing aggressive interactions without the opportunity to escape is stressful for sows (as reviewed by [19]) and pre-slaughter stress has long been recognised as negative for the welfare [1,25] and meat quality of pigs [26]. Hence, if the present preliminary, descriptive data are confirmed by larger, more controlled studies, the findings may suggest that a stay in a transfer vehicle before being transported to an abattoir may add additional stress to cull sows as compared to sows waiting on the farm. There are, however, no studies focusing on the behaviour of sows during transport, and the available studies on market pigs have shown conflicting results regarding the proportion and time development in resting and active behaviour during transport (as discussed by [27]). Hence, at present, it is not known whether a stay in a transfer vehicle is comparable, in terms of animal welfare, to being on a commercial truck during driving. Therefore, further research is needed in order to determine the welfare consequences of the use of transfer vehicles as compared to animals being picked up on the farm. In any case, the current results question whether the practice of using transfer vehicles follow recent recommendations from the OIE [12] underlining the possible vulnerability of cull sows and stating that, for example, old, lactating and aggressive animals require special conditions on the day of slaughter in order to ensure their welfare. 

## 5. Conclusions

In conclusion, this study is the first to describe the behaviour of cull sows while waiting in a transfer vehicle. The preliminary results suggest that aggressive interactions are common and that resting is limited, which might render a stay in a transfer vehicle before transport to the abbatoir a challenge for cull sow welfare, especially for longer stays and during hot days.

## Figures and Tables

**Table 1:** Description of the behaviour of cull sows from 11 loads from six commercial Danish sow herds while waiting in transfer vehicles before transport to the abattoir. In addition, the observation date (Obs. Date), herd ID, duration of the stay in the vehicles, stocking density, provision of bedding (sawdust), the presence of a roof on the vehicles (yes/no), access to water (yes/no) and mean temperatures (Temp.) are shown.

Obs. Date	Herd ID	Cull Sows	sow/m^2^	Duration, min	Occurrence. Lying	Freq. Lying/min	Occurrence Aggression	Freq. Aggression/Min	Bedding	Roof on Vehicle	Water Available	Mean Temp., °C
22 January	3	10	0.5	6	0	0	4	0.67	Yes	Yes	No	
28 January	4	8	0.5	8	0	0	6	0.75	Yes	No	No	
2 February	5	9	1.2	56	0	0	65	1.16	No	No	No	
9 March	5	9	1.2	6	0	0	5	0.83	No	No	No	10.6 ± 0.8
13 April	5	9	1.2	15	1	0.07	16	1.07	No	No	No	
2 July	2	12	0.6	58	23	0.40	38	0.66	.	No	No	26.3 ± 1.2
8 July	1	12	1.7	32	1	0.04	23	0.72	No	No	No	19.6 ± 0.4
19 August	1	7	0.6	53	10	0.19	47	0.89	No	No	No	20.7 ± 0.5
2 September	1	13	1.8	59	4	0.07	29	0.49	No	No	No	15.0 ± 0.8
16 September	1	10	1.4	19	4	0.21	18	0.95	No	No	No	13.1 ± 1.2
14 December	6	7	0.4	21	6	0.29	15	0.71	.	Yes	No	5.3 ± 0.3
**Median**		**9**	**1.2**	**21**	**1**	**0.07**	**18**	**0.75**				
